# Supplementary material for: Heimdallarchaea encodes profilin with eukaryotic-like actin regulation and polyproline binding
Source: Commun Biol. 2021 Sep 1;4:1024. doi: 10.1038/s42003-021-02543-x (PMC8410842; doi:10.1038/s42003-021-02543-x)
Supplement: Supplementary file 2 — Supplementary Information [file 42003_2021_2543_MOESM2_ESM.pdf]

Heimdallarchaea encodes profilin with eukaryotic-like actin regulation and polyproline binding

Sabeen Survery[1*], Fredrik Hurtig[1], Syed Razaul Haq[1], Jens Eriksson[2], Lionel Guy[2], K. Johan Rosengren[3], Ann-Christin Lindås[1], and Celestine N. Chi[2] *.

[1]Department of Molecular Bioscience, The Wenner-Gren Institute, Stockholm University, Svante Arrhenius v. 20C, SE-10691 Stockholm, Sweden

[2]Department of Medical Biochemistry and Microbiology, Uppsala University, BMC Box 582, SE-75123 Uppsala, Sweden.

[3]School of Biomedical Sciences, The University of Queensland, Brisbane, QLD 4072, Australia

*Corresponding authors: sabeen.survery@su.se and chi.celestine@imbim.uu.se

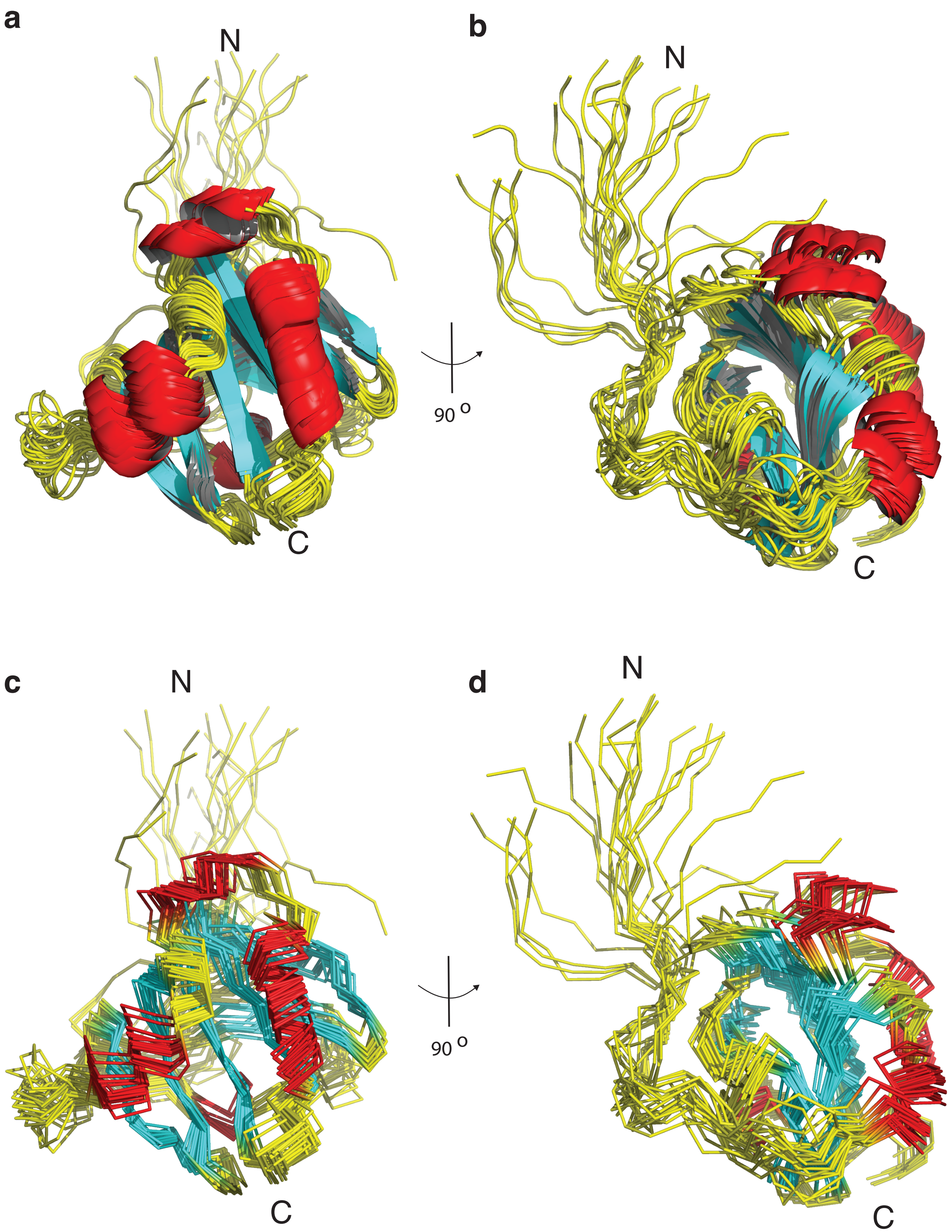

Supplementary Figure 1. Structural representation of profilin. a, Ensemble of the 20 structures of heimProfilin shown as cartoon. **b**, Orientation of the structures in (a) showing the N-terminal

extension. **c,** Same as (a) but displayed in lines. **d**, Same as (b) but displayed in lines. The coordinates have been deposited in the Protein databank with PDB ID: 6YRR

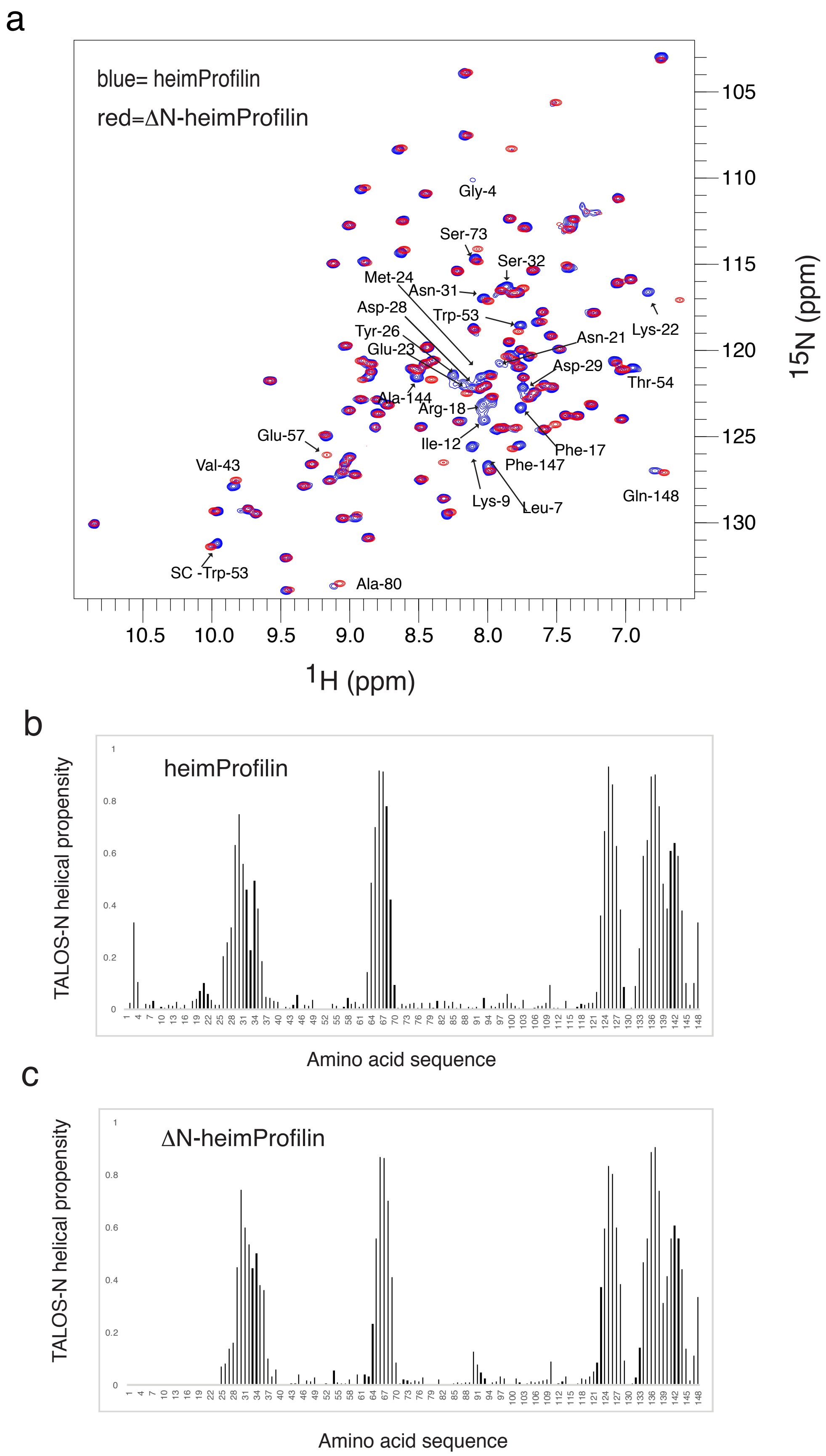
a
blue= heimProfilin
red=ΔN-heimProfilin
Gly-4
Ser-73
Ser-32
Met-24
Asn-31
Trp-53
Asp-28
Tyr-26
Glu-23
Asn-21
Lys-22
Asp-29
Thr-54
Ala-144
Arg-18
Glu-57
Ile-12
Phe-17
Phe-147
Val-43
Lys-9
Leu-7
Gln-148
SC -Trp-53
Ala-80
1H (ppm)
15N (ppm)
b
heimProfilin
TALOS-N helical propensity
Amino acid sequence
c
ΔN-heimProfilin
TALOS-N helical propensity
Amino acid sequence

9

Supplementary Figure 2. ^{1}H-^{15}N TROSY HSQC spectra of heimProfilin and ΔN-heimProfilin and helical propensity. a, Overlay of HSQC spectra of heimProfilin (blue) and ΔN-heimProfilin (red). The residues from the N-terminal extension are labeled, including those that underwent slight chemical shift changes. Overall, the resonances of most residues overlay between both proteins indicating little or no structural change. TALOS-N helical propensity predicted from backbone chemical shifts plotted as a function of amino acid sequence for heimProfilin (**b**) and ΔN-heimProfilin (**c**)**.** Regions with consecutive bars indicate high helical content. Overall these data together with the ^{1}H-^{15}N chemical shift indicate that the overall structure of heimProfilin and ΔN-heimProfilin are similar.

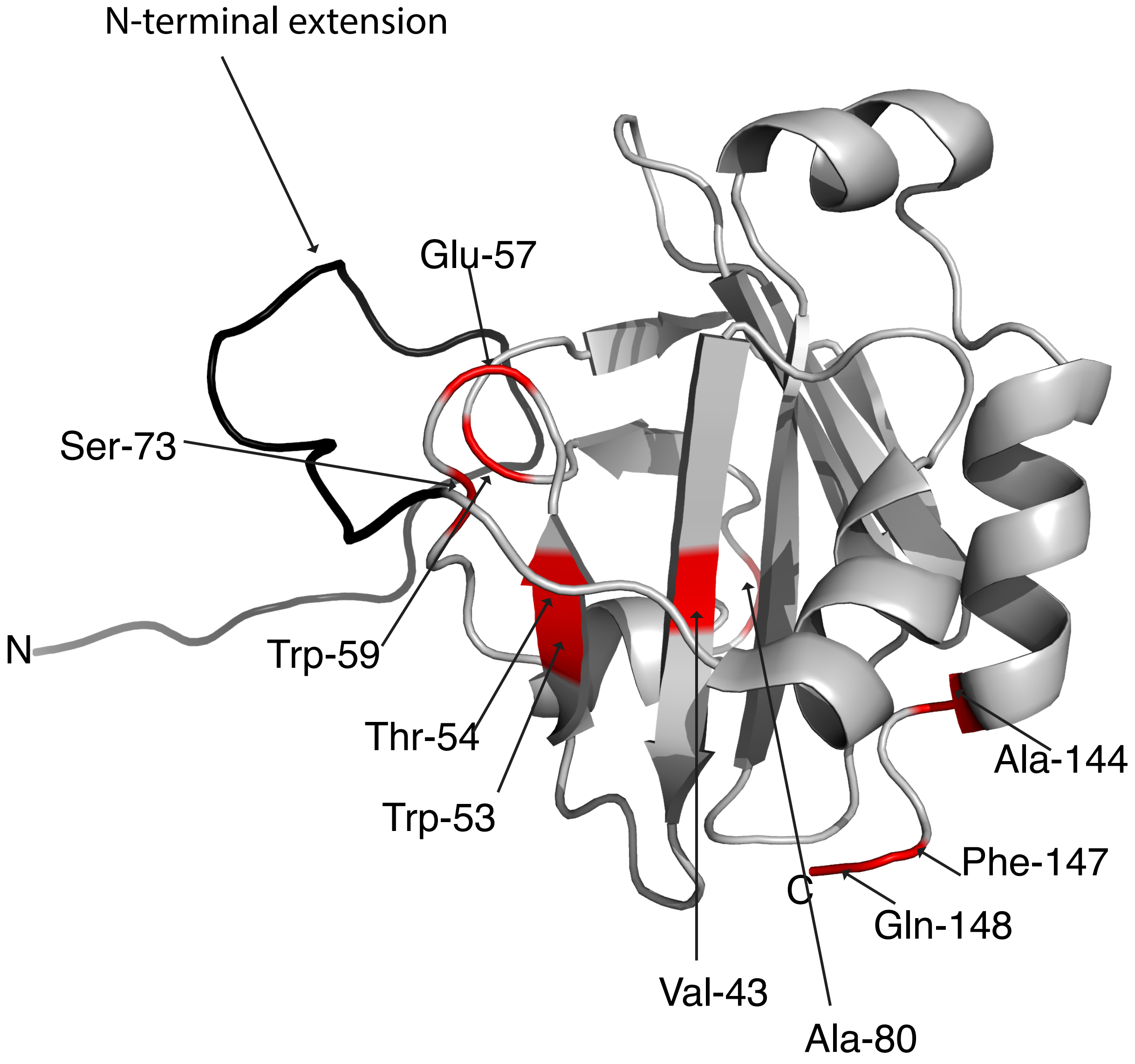

Supplementary Figure 3. Structural representation of chemical shift changes upon mutation. Residues that displayed a change in their ^{1}H-^{15}N shift upon deletion of the N-terminal extension are color coded on the structure of heimProfilin. The N-terminal extension is also indicated.

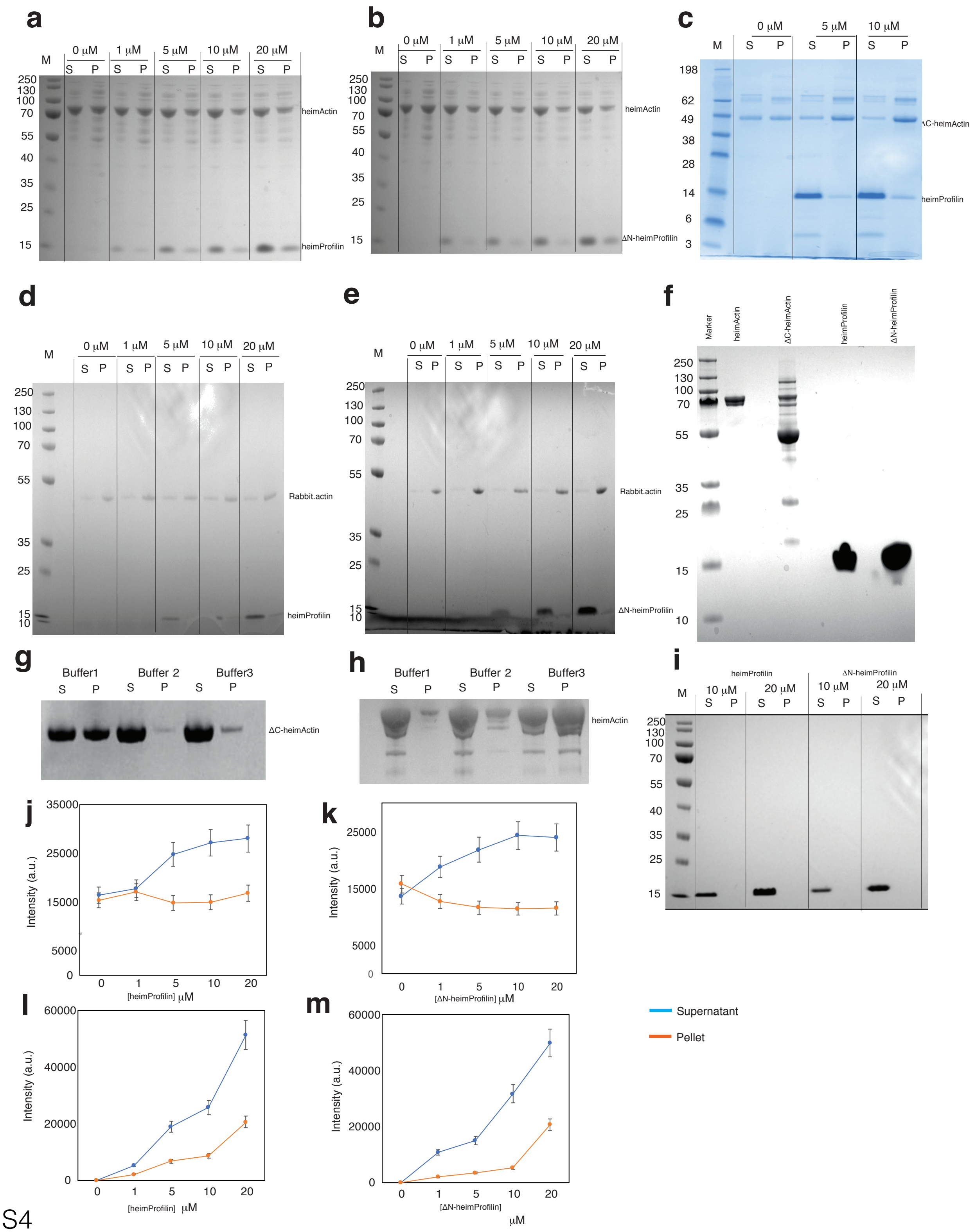

S4

Supplementary Figure 4. Purification of Heimdallarchaeota actin and profilin and rabbit actin sedimentation. a, Sedimentation assay for heimActin alone and with increasing

concentrations of heimProfilin. The amount of heimProfilin in the pellet (p) increases with concentration **b**, Same as in (a) but with heimActin and ΔN-heimProfilin. Here, both ΔN-heimProfilin and heimActin appear in the soluble fraction (s) and the pellet, indicating that ΔN-heimProfilin interacts with actin monomer as well as polymerized heimActin. **c**, Sedimentation of ΔC-heimActin with different concentrations of heimProfilin. Here, heimProfilin is found in both the soluble and pellet fraction indicating interaction between ΔC-heimActin and heimProfilin. **d,** Same as in (a) but with rabbit actin and **e,** same as in (b) but with rabbit actin. **f**, SDS-PAGE of purified ΔC-heimActim, heimActin, heimProfilin and ΔN-heimProfilin. **g,** and **h,** Polymerization buffer screening for the ΔC-heimActin and heimActin. Both supernatant (s) and pellet (p) after ultracentrifuge were loaded onto an SDS-PAGE gel to select the optimal buffer for polymerization. ΔC-heimActin showed the optimal polymerization in buffer 1 (2 mM Tris-HCl pH 7.5, 100 mM KCl, 1 mM ATP, 2 mM $MgCl_2$, 10 mM imidazol) while buffer 3 (20 mM Tris-HCl pH 8.0, 250 mM KCl, 1 mM ATP, 4 mM $MgCl_2$) was optimal for heimActin. **i,** Sedimentation profiles of free heimProfilin and ΔN-heimProfilin at two different concentrations. No protein was detected in the pellet fraction in both cases. This indicates that heimProfillin and ΔN-heimProfilin are soluble and the bands detected in the sedimentation with actin is due to its interaction with the respective actin. **i-m,** Dosimetry quantification of the co-sedimentation assays in (a) and (b) between heimProfilin and heimActin. There is a gradual decrease of heimActin in the pellet (p) while the concentration in the supernatant (s) increases with increasing concentration of heimProfilin (j). Similar trends are observed for the co-sedimentation between ΔN-heimProfilin and heimActin with increasing fraction of heimaActin in the supernatant (k). Increasing the concentration of heimProfilin and ΔN-heimProfilin increases the concentrations in the both the pellet and supernatant (l and m). 5 μM actin was used in above assays.

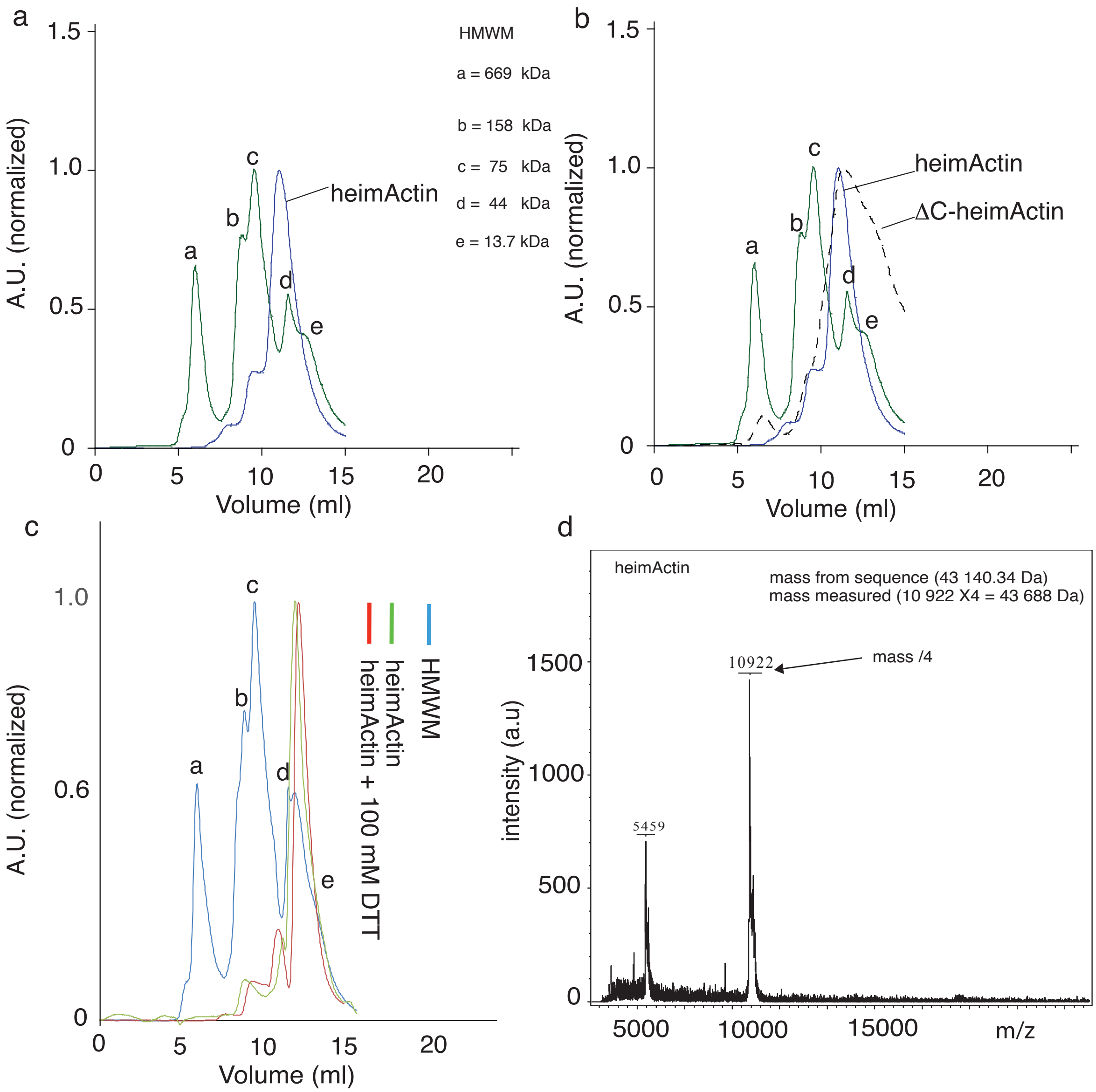

e

```
Heimda_LC3 MEEMIQNKPLVIDNGTGFTKNGFAGEDQPRSVFPTIIGYPKYQIIMTDVEHYVREYYIGE 60
Heimda_125 MEEMIQNKPLVIDNGTGFTKNGFAGEDQPRSVFPTIIGYPKYQIIMTDVEHYVREYYIGE 60
           ************************************************************

Heimda_LC3 EAINLRGVLKLVYPVEHGQVQDWDAMERIWHYTFYNDLRVNPNEHPVLLTEPPLNKNQNK 120
Heimda_125 EAINLRGVLKLVYPVEHGQVQDWDAMERIWHYTFYNDLRVNPNEHPVLLTEPPLNKNQNK 120
           ************************************************************

Heimda_LC3 EKMAELMFDTFNVPAMYISMQAILSLYASGRTTGIVVDSGDGVTHIVPVYEGFAISHAIH 180
Heimda_125 EKMAELMFDTFNVPAMYISMQAILSLYASGRTTGIVVDSGDGVTHIVPVYEGFAISHAIH 180
           ************************************************************

Heimda_LC3 RSDIGGRDITDYLRRLLRQRGYSLSSSAEREIVRDIKERLCYVALDPEKELKLAEKVSGM 240
Heimda_125 RSDIGGRDITDYLRRLLRQRGYSLSSSAEREIVRDIKERLCYVALDPEKELKLAEKVSGM 240
           ************************************************************

Heimda_LC3 EKTYTLPDGETLTIGAERFMAPELTFNPGAIGSEENPLDELIYRSIQNCDVDLRRDLYAN
Heimda_125 EKTYTLPDGETLTIGAERFMAPELTFNPGAIGSEENPLDELIYRSIQNCDVDLRRDLYAN
           ************************************************************

Heimda_LC3 IVLSGGSTMFPGLKERLHKELTELVPETMEVKIIAPPERRYSVWIGGSILSSLKTFAKLW 360
Heimda_125 IVLSGGSTMFPGLKERLHKELTELVPETMEVKIIAPPERRYSV----------------- 343
           *******************************************

Heimda_LC3 VTRKEYREIGPTSVYRCI      378
Heimda_125 ------------------      343
```

51 S.5

Supplementary Figure 5. Analytical size exclusion chromatography of purified heimActin. Analytical size exclusion chromatography of heimActin compared with high molecular weight markers(a-e). **a**, heimActin (43140.34 Da) elutes at the same retention time as 44 kDa molecular weight standard. **b**, Elution profile of heimActin and ΔC-heimActin plotted as a function of the high molecular weight standards (HMWM). **c**, elution of profile of heimActin in the presence (red) or absence (green) of 100 mM DTT as a function of the high molecular weight standards. **d**, MALDI-TOF mass spectrometry of purified heimActin. heimActin could be detected at mass/charge ratio of mass/4. e, sequence alignment of Heimdallarchaeota LC3 and AB_125. Th C-terminal tail is highlighted in yellow.

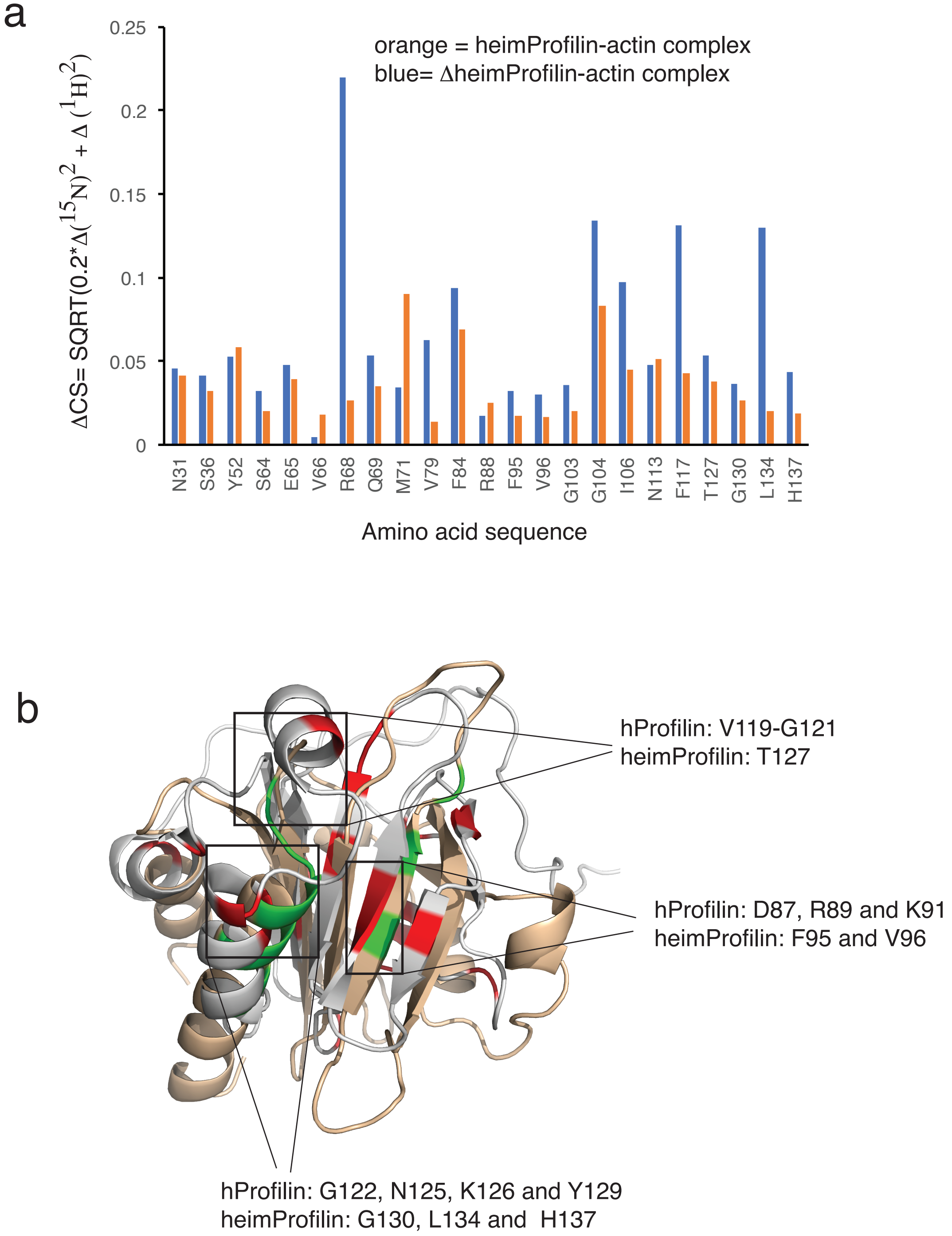

Supplementary Figure 6. Plots of the chemical shift changes of heimProfilin and ΔN-heimProfilin interacting with heimActin. a, Chemical shift plotted as histogram as a function of amino acids for heimProfilin (orange) and ΔN-heimProfilin (blue). Only those residues experiencing shift are plotted. **b**, Overlay of structures of heimProfilin (grey) and human profilin (gold) displaying residues/regions required for actin binding in heimProfilin (red) and human

profilin (green).

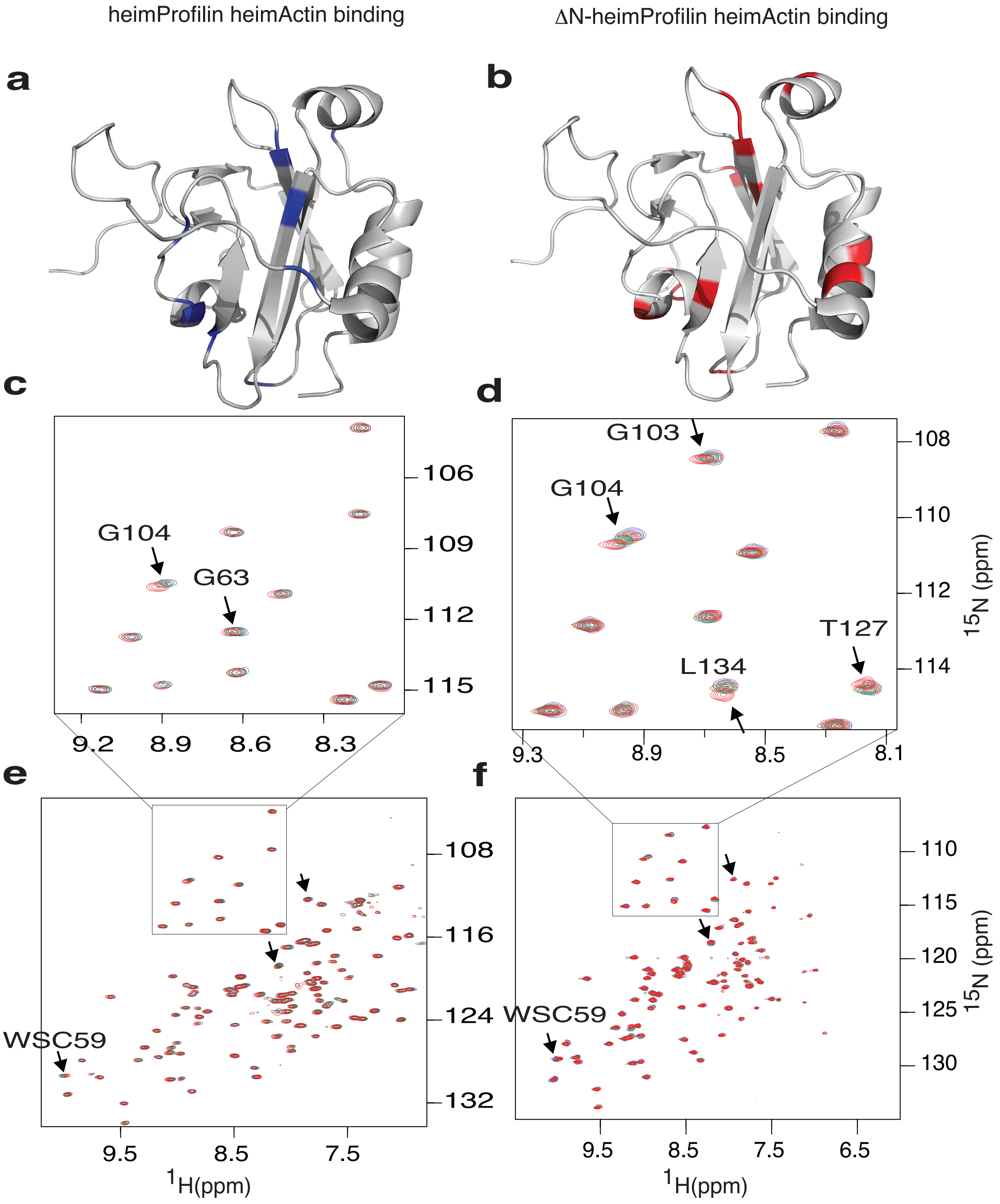

Supplementary Figure 7. Nuclear magnetic resonance binding interaction of Heimdallarchaeota actin and heimProfilin. Schematic of the structure of heimProfilin showing interacting regions for **a**, heimActin with heimProfilin (blue). **b**, heimActin with ΔN-heimProfilin

(red) (mapped onto structure of heimProfilin) **c,** Expansion of overlay ^{1}H-^{15}N HSQC spectra showing chemical shift changes for some of the residues for heimProfilin (100 µM) with increasing concentrations of heimActin; and 0 µM (red), 80 µM (green) and 200 µM (green). **d**, Expansion of overlay ^{1}H-^{15}N HSQC spectra showing chemical shift changes for some of the residues for ΔN-heimProfilin (100 µM) with increasing concentrations of heimActin (0 µM (red), 20 µM (cyan), 80 µM (magenta), 120 µM (green) and 200 µM (blue). **e,** Overlay of ^{1}H-^{15}N HSQC spectra for heimProfilin. **f,** Overlay of ^{1}H-^{15}N HSQC spectra for ΔN-heimProfilin with increasing concentrations of heimActin. Color codes are as stated above. Note, that not all residues experience chemical shift changes as indicated with the arrows in (e) and (f).

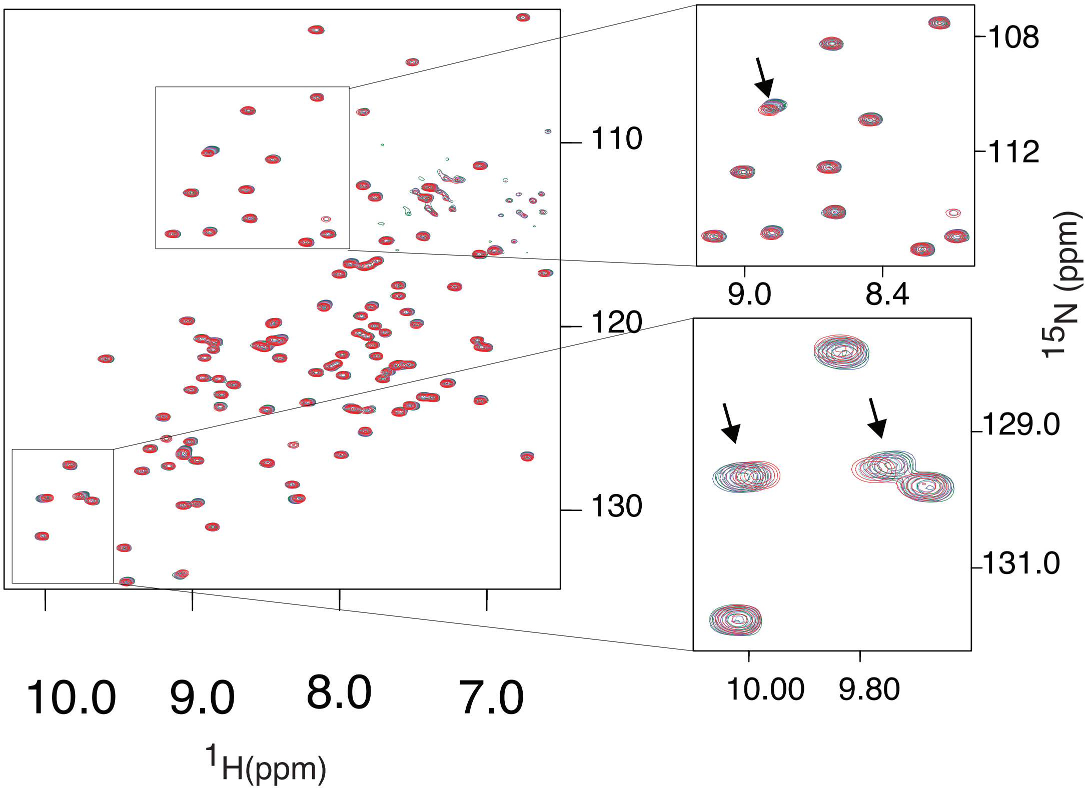

Supplementary Figure 8. Nuclear magnetic resonance binding interaction of Heimdallarchaeota; ΔC-heimActin and ΔN-heimProfilin. Overlay of ^{1}H-^{15}N HSQC spectra of ΔN-heimProfilin (100 µM) with increasing concentrations of ΔC-heimActin (0 µM (red), 20 µM (cyan), 80 µM (magenta), 120 µM (green) and 200 µM (blue).

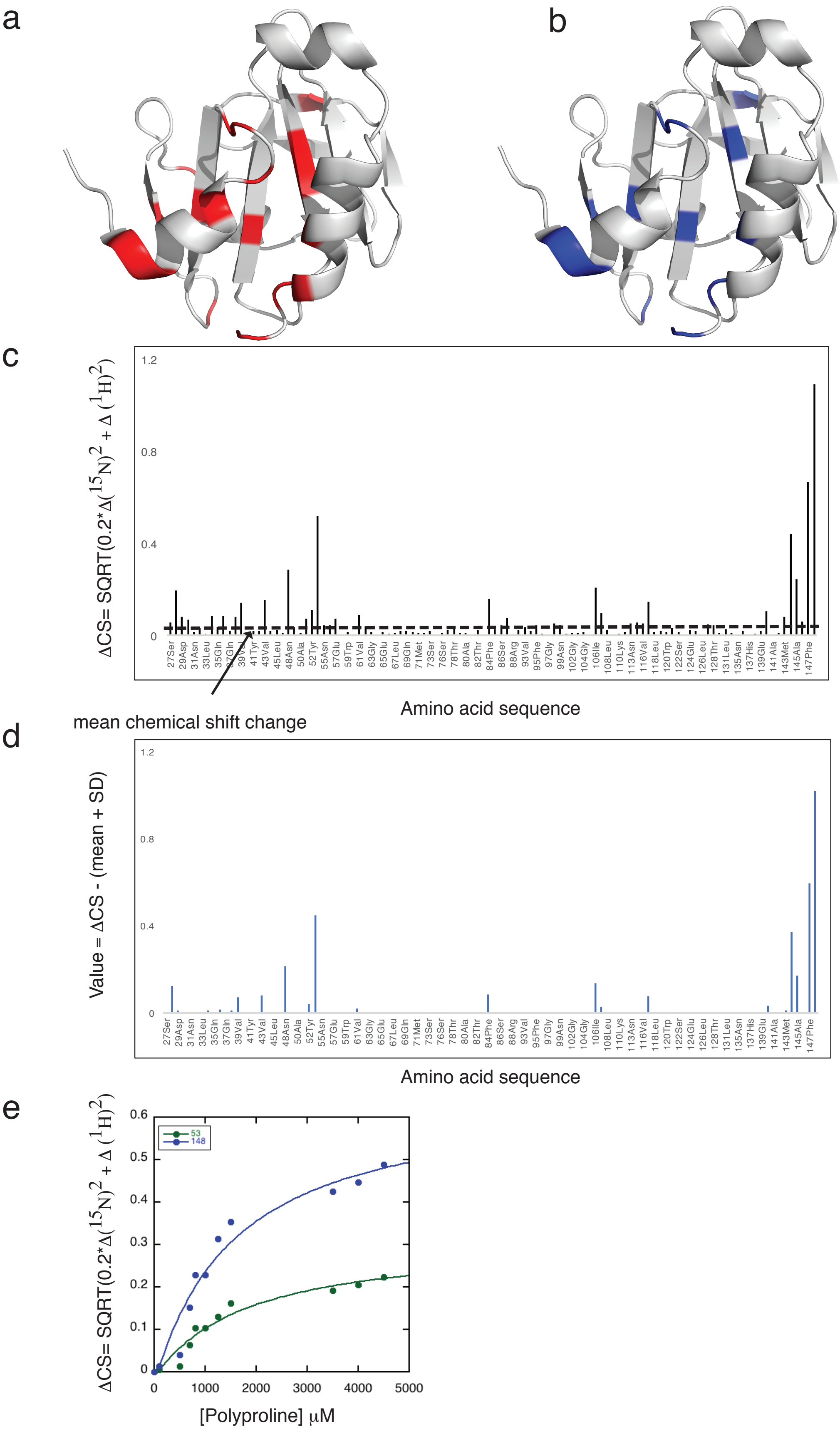
a
b
c
ΔCS= SQRT(0.2*Δ(^{15}N)^2 + Δ (^{1}H)^2)
1.2
0.8
0.4
0
27Ser 29Asp 31Asn 33Leu 35Gln 37Gln 39Val 41Tyr 43Val 45Leu 48Asn 50Ala 52Tyr 55Asn 57Glu 59Trp 61Val 63Gly 65Glu 67Leu 69Gln 71Met 73Ser 76Ser 78Thr 80Ala 82Thr 84Phe 86Ser 88Arg 93Val 95Phe 97Gly 99Asn 102Gly 104Gly 106Ile 108Leu 110Lys 113Asn 116Val 118Leu 120Trp 122Ser 124Glu 126Leu 128Thr 131Leu 135Asn 137His 139Glu 141Ala 143Met 145Ala 147Phe
Amino acid sequence
mean chemical shift change
d
Value = ΔCS - (mean + SD)
1.2
0.8
0.4
0
27Ser 29Asp 31Asn 33Leu 35Gln 37Gln 39Val 41Tyr 43Val 45Leu 48Asn 50Ala 52Tyr 55Asn 57Glu 59Trp 61Val 63Gly 65Glu 67Leu 69Gln 71Met 73Ser 76Ser 78Thr 80Ala 82Thr 84Phe 86Ser 88Arg 93Val 95Phe 97Gly 99Asn 102Gly 104Gly 106Ile 108Leu 110Lys 113Asn 116Val 118Leu 120Trp 122Ser 124Glu 126Leu 128Thr 131Leu 135Asn 137His 139Glu 141Ala 143Met 145Ala 147Phe
Amino acid sequence
e
ΔCS= SQRT(0.2*Δ(^{15}N)^2 + Δ (^{1}H)^2)
53
148
0.6
0.5
0.4
0.3
0.2
0.1
0
0
1000
2000
3000
4000
5000
[Polyproline] μM

Supplementary Figure 9. Chemical shift (CS) perturbation of the interaction of between ΔN-heimProfilin and polyproline (*PPPAPPLPAAQ*). Structures of ΔN-heimProfilin displaying the interaction sites of polyproline. **a,** Residues displaying chemical shifts above mean value are color coded in red**. b,** Residues displaying chemical shift above mean + 1 standard deviation are color coded in blue **c,** Chemical shifts plotted as function of amino acid sequence. Shifts were obtained by taking the difference between the free and bound protein. The dotted line indicates the mean chemical shift change. A few residues displayed significant difference from the mean which represent ΔN-heimProfilin:polyproline interaction. **d**, Chemical shift, reduced by mean + 1 standard deviation, plotted as function of amino acid sequence. **e,** Chemical shift plotted as function of polyproline concentration for a few residues. The chemical shift was fitted to the equation describing the equilibrium interaction of two biomolecules (CS = [($[P]_0$ +K_D + n)/2 – SQRT ($[P]_0$ +K_D + n)$^{\wedge 2}$ /4 -($[P]_0 n$] x B + C; CS is chemical shift difference, $[P]_o$ and n are the total concentrations of the varied and non-varied species respectively, K_D is the equilibrium dissociation constant and B and C are constants that take into consideration the total signal change and the signal at ($[P]_0 = 0$), respectively. The titrations were done so that the total volume in the NMR tube and hence the concentrations of protein change, was minimal. The K_D estimated from this range between 1.6 -1.8 ± 0.6 mM. The K_D estimated from chemical shift are generally higher than those from other methods because of a number of uncertainties.

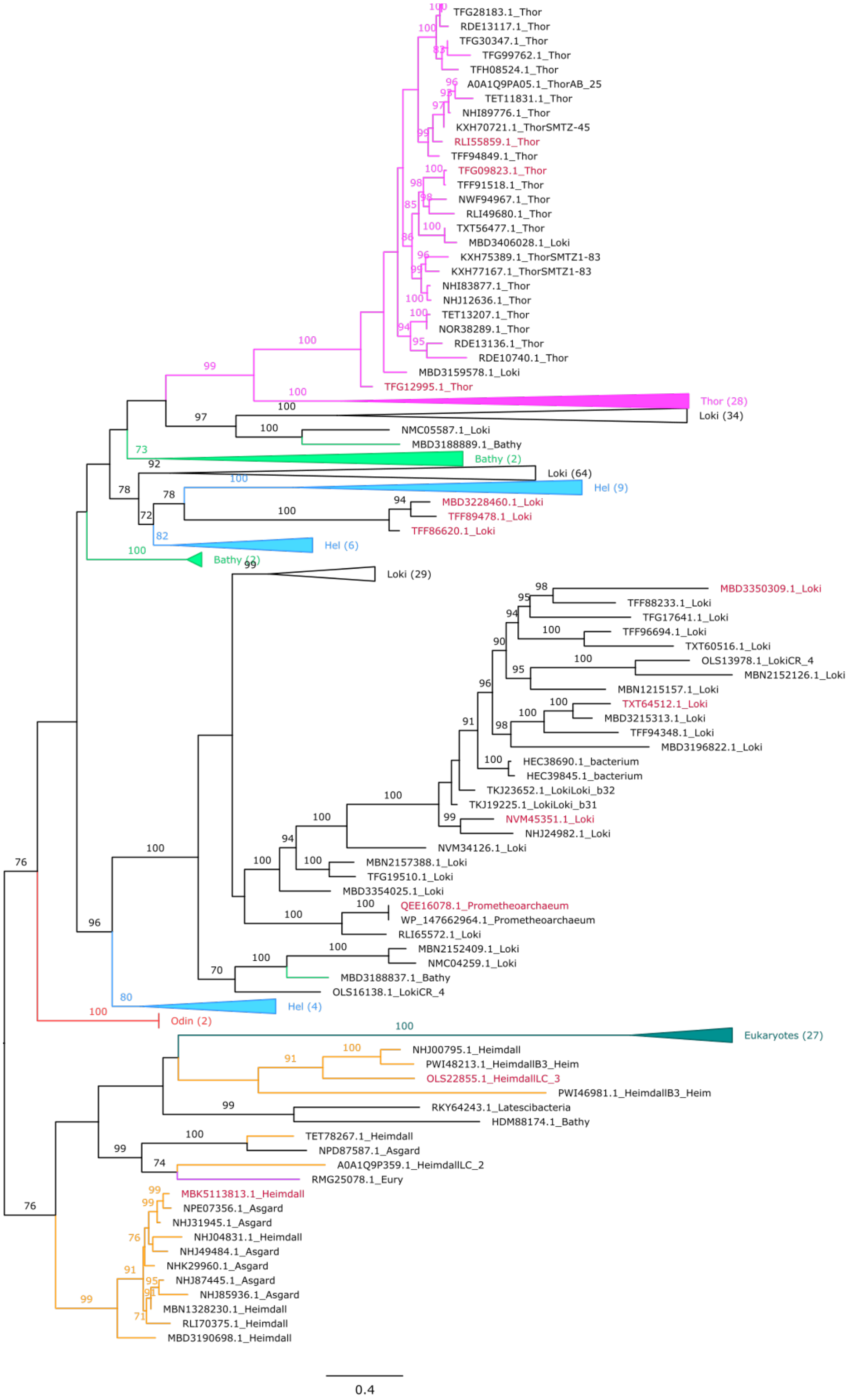

Supplementary Figure 10: Maximum-likelihood phylogeny of the archaeal profilin homologs.

The tree is based on a trimmed alignment of 290 profilin homologs (including 27 eukaryotic sequences) gathered with PSI-BLAST, starting from a heimProfilin (OLS22855.1). The tree is arbitrarily rooted with Heimdallarchaeota. The accession number of the protein is suffixed with its taxonomic affiliation. Proteins with a N-terminal extension longer than 5 amino-acid residues are shown with a red font. Groups of homogenous taxonomic content and not containing extensions were collapsed for clarity. The number of sequences in each collapsed group is given between parentheses. Branch colors represent the different groups of Archaea: Thorachaeota (pink), Lokiarchaeota (black), Helarchaeota (blue), Euryarchaeota (purple), and Bathyarchaeota (green). Eukaryotes are shown in dark green. The scale represents the average number of substitutions per site. Numbers on branches are the percent of bootstrap support for that branch. Bootstrap support lower than 70 is not displayed.

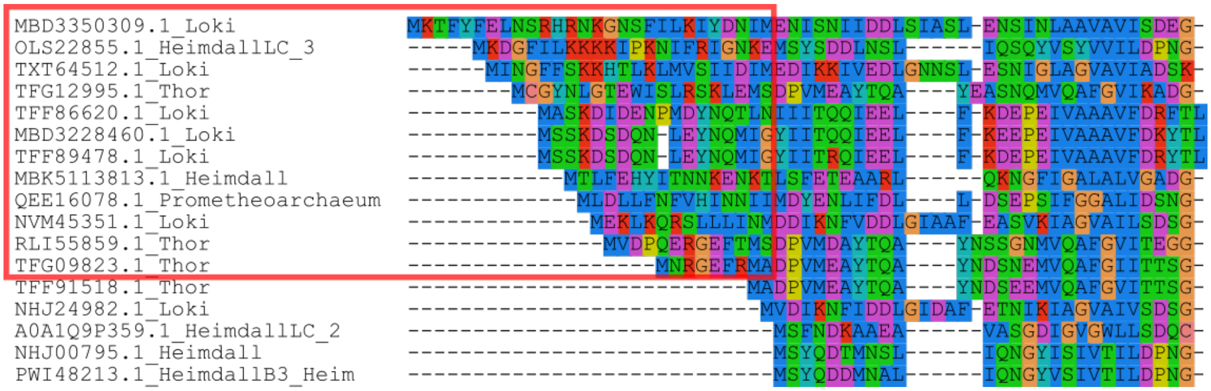

Supplementary Figure 11: Multiple sequence alignment of profilin homologs and their N-terminal extensions. The N-terminal extension is represented in the red box. The sequences below are shown for comparison. The extension is not aligned and is presented gapless, except for the three related Lokiarchaeota sequences (TFF86620.1, TFF89478.1, MBD3228460.1). Only the start of the alignment is shown.

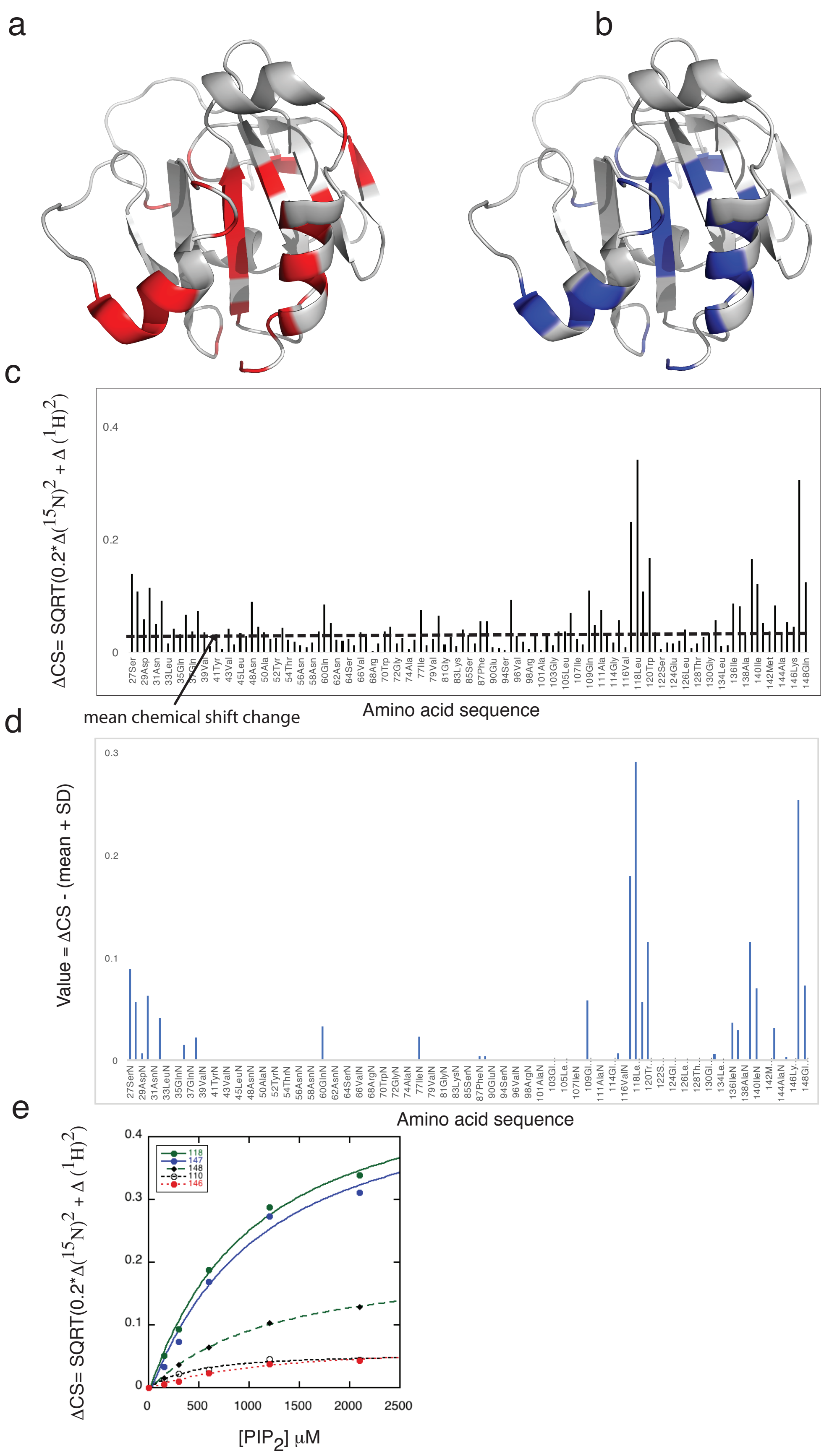
a
b
c
ΔCS= SQRT(0.2*Δ(15N)2 + Δ (1H)2)
0.4
0.2
0
mean chemical shift change
Amino acid sequence
d
Value = ΔCS - (mean + SD)
0.3
0.2
0.1
0
Amino acid sequence
e
ΔCS= SQRT(0.2*Δ(15N)2 + Δ (1H)2)
118
147
148
110
146
0.4
0.3
0.2
0.1
0
0
500
1000
1500
2000
2500
[PIP2] μM

141

Supplementary Figure 12. Structural representation of heimProfilin showing PIP_2 binding residues. a, Cartoon representation of heimProfilin for residues displaying chemical shift above the mean on interaction with PIP_2 (red). **b,** Cartoon representation of heimProfilin for residues displaying chemical shift above the mean + 1 standard deviation on interaction with PIP_2 (blue). **c,** Chemical shift plotted as function of amino acid sequence. Chemical shift was obtained by taking the difference between the free and bound protein. The dotted line indicates the mean chemical shift change. A few residues had significant difference from the mean and represent protein-PIP_2 binding. **d**, Chemical shift, reduced by mean + 1 standard deviation, plotted as function of amino acid sequence. **e,** Chemical shift plotted as function of PIP_2 concentration for a few residues. The chemical shift was fitted to the equation describing the equilibrium interaction of two biomolecules as described above. The K_D estimated from this fits range between 300-600 μM.

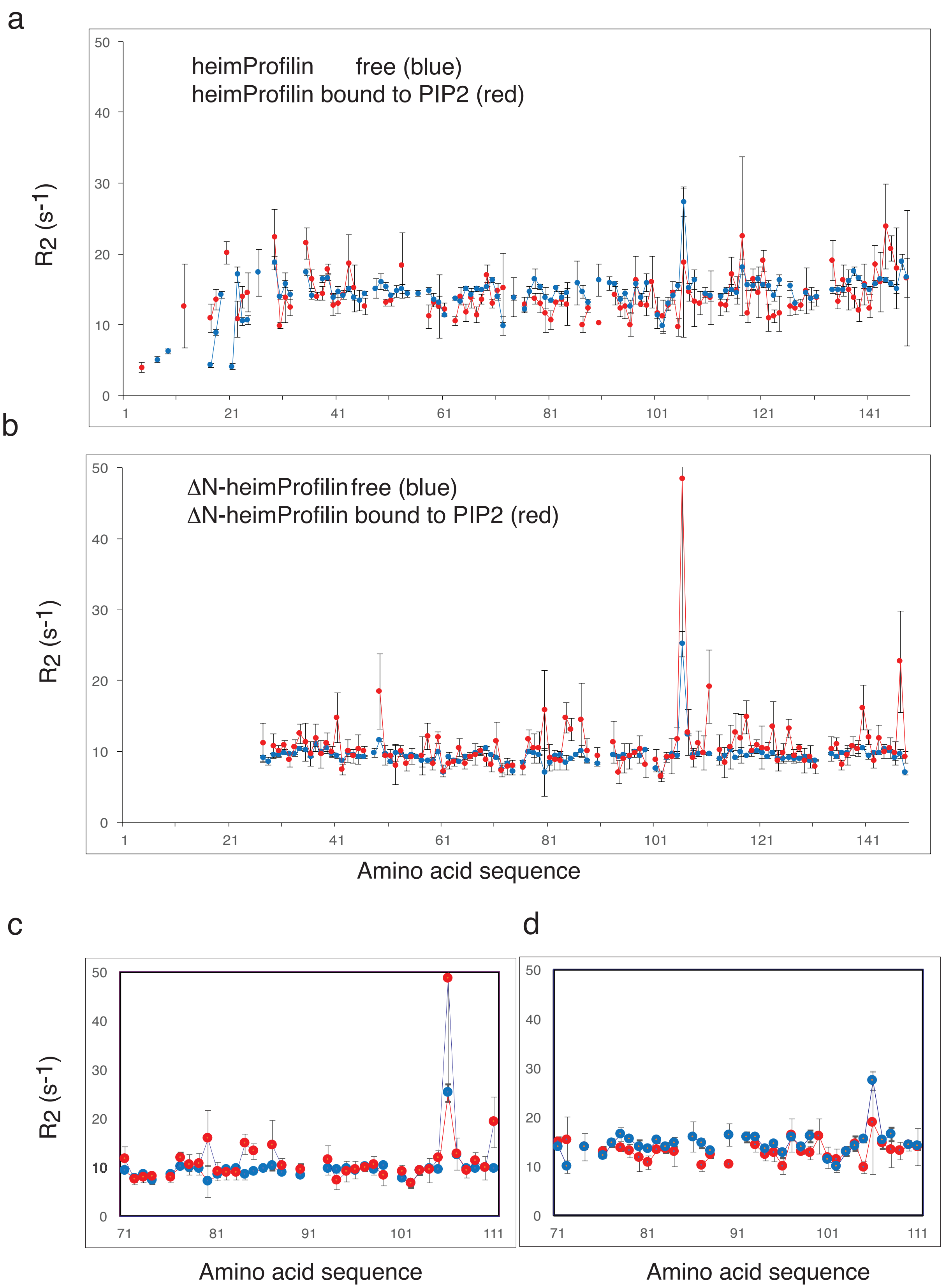

Supplementary Figure 13. Relaxation rates (*R_2*) for free and bound heimProfilin with PIP_2.

a, R_2 rates plotted as a function of amino acid sequence for heimProfilin free (blue) and bound to PIP_2 (red). **b,** R_2 rates plotted as a function of amino acid sequence for ΔN-heimProfilin free (blue) and bound to PIP_2 (red). **c**, Expansion of a region in (b). **d**, Expansion of a region in (a). Increase in R_2 rates is attributed to contribution from R_{ex} as a result of motions in the sub-millisecond range.

a

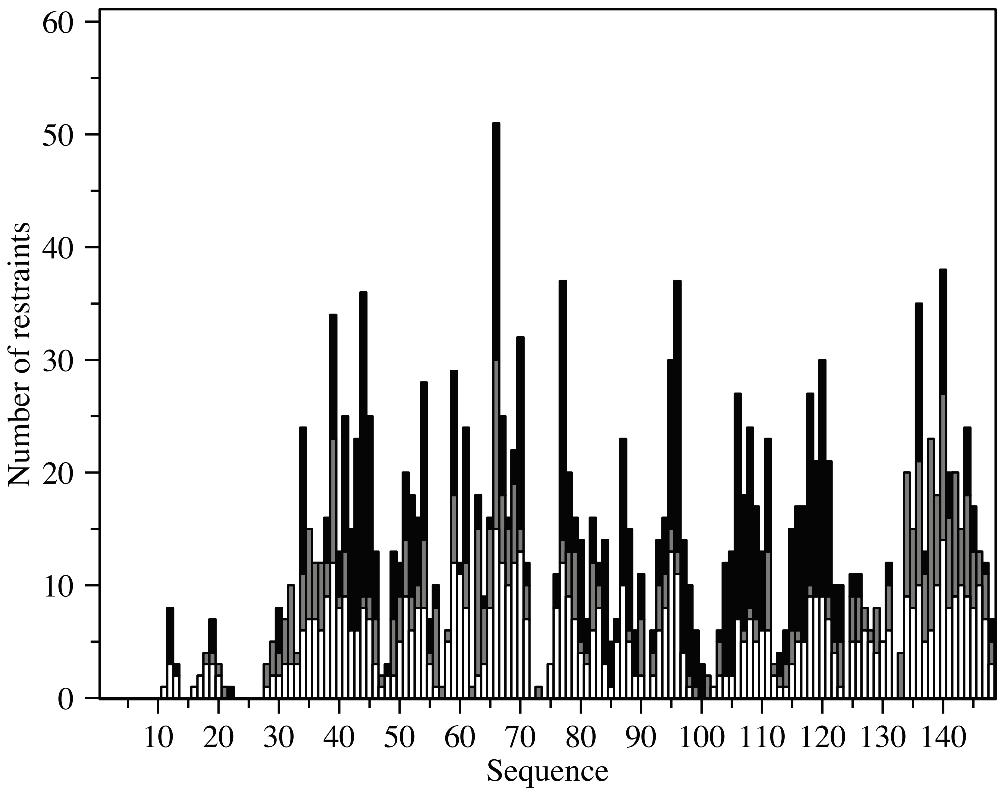
Number of restraints
0
10
20
30
40
50
60
10 20 30 40 50 60 70 80 90 100 110 120 130 140
Sequence

b

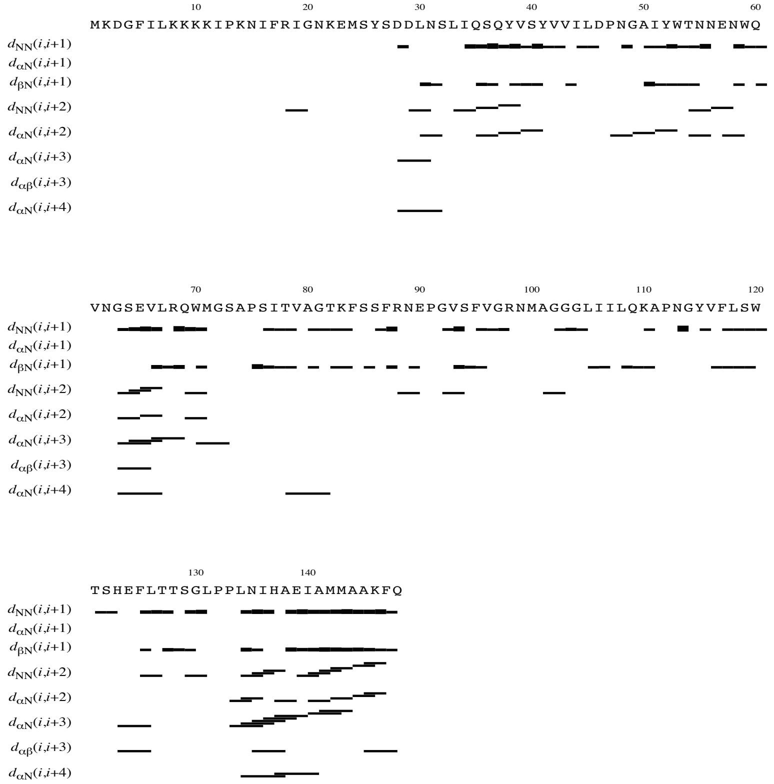
10 20 30 40 50 60
MKDGFILKKKKIPKNIFRIGNKEMSYSDDLNSLIQSQYVSYVVILDPNGAIYWTNNNENWQ
70 80 90 100 110 120
VNGSEVLRQWMGSAPSITVAGTKFSSFRNEPGVSFVGRNMAGGGLIILQKAPNGYVFLSW
130 140
TSHEFLTTSGLPPLNIHAEIAMMAAKFQ
dNN(i,i+1)
dαN(i,i+1)
dβN(i,i+1)
dNN(i,i+2)
dαN(i,i+2)
dαN(i,i+3)
dαβ(i,i+3)
dαN(i,i+4)

Supplementary Figure 14. NMR NOE restraints. a, Total number of restraints plotted as function of amino acid sequence**. b,** Medium and long NOE restraints plotted as lines as a function of amino acid sequence**.**
