# Supplementary material for: Heimdallarchaea encodes profilin with eukaryotic-like actin regulation and polyproline binding
Source: Commun Biol. 2021 Sep 1;4:1024. doi: 10.1038/s42003-021-02543-x (PMC8410842; doi:10.1038/s42003-021-02543-x)
Supplement: Supplementary file 3 — Description of Additional Supplementary Files [file 42003_2021_2543_MOESM3_ESM.pdf]

Description of Additional Supplementary Files

File name: Supplementary Movie 1

Description: Rabbit actin polymerization in the presence of different profilins and mutants

File name: Supplementary Data 1

Description: Detailed heimProfilin structure refinement statistics from CNS
